# Trends in research on pain relief during oocyte retrieval for IVF/ICSI: a systematic, methodological review

**DOI:** 10.1093/hropen/hoac006

**Published:** 2022-02-16

**Authors:** E T I A Buisman, H Grens, R Wang, S Bhattacharya, D D M Braat, A G Huppelschoten, J W van der Steeg

**Affiliations:** 1 Department of Obstetrics and Gynaecology, Jeroen Bosch Ziekenhuis, ‘s-Hertogenbosch, The Netherlands; 2 Department of Obstetrics and Gynaecology, Radboud University Medical Centre, Nijmegen, The Netherlands; 3 Department of Obstetrics and Gynaecology, Monash University, Clayton, Victoria, Australia; 4 Department of Obstetrics and Gynaecology, Aberdeen Maternity Hospital, Aberdeen, UK; 5 Department of Obstetrics and Gynaecology, Catharina Hospital, Eindhoven, The Netherlands

**Keywords:** methodology / pain / analgesia / oocyte retrieval / IVF/ICSI / reporting / randomized controlled trials / core outcome sets

## Abstract

**STUDY QUESTION:**

What is the methodological validity and usefulness of randomized controlled trials (RCTs) on pain relief during oocyte retrieval for IVF and ICSI?

**SUMMARY ANSWER:**

Key methodological characteristics such as randomization, allocation concealment, primary outcome measure and sample size calculation were inadequately reported in 33–43% of the included RCTs, and a broad heterogeneity is revealed in the studied outcome measures.

**WHAT IS KNOWN ALREADY:**

A Cochrane review on conscious sedation and analgesia for women undergoing oocyte retrieval concluded that the overall quality of evidence was low or very low, mainly owing to poor reporting. This, and heterogeneity of studied outcome measures, limits generalizability and eligibility of results for meta-analysis.

**STUDY DESIGN, SIZE, DURATION:**

For this review, a systematic search for RCTs on pain relief during oocyte retrieval was performed on 20 July 2020 in CENTRAL CRSO, MEDLINE, Embase, PsycINFO, CINAHL, ClinicalTrials.gov, WHO ICTRP, Web of Science, Portal Regional da BVS and Open Grey.

**PARTICIPANTS/MATERIALS, SETTING, METHODS:**

RCTs with pain or patient satisfaction as an outcome were included and analysed on a set of methodological and clinical characteristics, to determine their validity and usefulness.

**MAIN RESULTS AND THE ROLE OF CHANCE:**

Screening of 2531 articles led to an inclusion of 51 RCTs. Randomization was described inadequately in 33% of the RCTs. A low-risk method of allocation concealment was reported in 55% of the RCTs. Forty-nine percent of the RCTs reported blinding of participants, 33% of blinding personnel and 43% of blinding the outcome assessor. In 63% of the RCTs, the primary outcome was stated, but a sample size calculation was described in only 57%. Data were analysed according to the intention-to-treat principle in 73%. Treatment groups were not treated identically other than the intervention of interest in 10% of the RCTs. The primary outcome was intraoperative pain in 28%, and postoperative pain in 2%. The visual analogue scale (VAS) was the most used pain scale, in 69% of the RCTs in which pain was measured. Overall, nine other scales were used. Patient satisfaction was measured in 49% of the RCTs, for which 12 different methods were used. Occurrence of side-effects and complications were assessed in 77% and 49% of the RCTs: a definition for these was lacking in 13% and 20% of the RCTs, respectively. Pregnancy rate was reported in 55% of the RCTs and, of these, 75% did not adequately define pregnancy. To improve the quality of future research, we provide recommendations for the design of future trials. These include use of the VAS for pain measurement, use of validated questionnaires for measurement of patient satisfaction and the minimal clinically relevant difference to use for sample size calculations.

**LIMITATIONS, REASONS FOR CAUTION:**

Consensus has not been reached on some methodological characteristics, for which we formulated recommendations. To prevent further heterogeneity in research on this topic, recommendations were formulated based on expert opinion, or on the most used method thus far. Future research may provide evidence to base new recommendations on.

**WIDER IMPLICATIONS OF THE FINDINGS:**

Use of the recommendations given for design of trials on this topic can increase the generalizability of future research, increasing eligibility for meta-analyses and preventing wastefulness.

**STUDY FUNDING/COMPETING INTEREST(S):**

No specific funding was obtained for this study. S.B. reports being the editor-in-chief of *Human Reproduction Open*. For this manuscript, he was not involved with the handling process within *Human Reproduction Open*, or with the final decision. Furthermore, S.B. reports personal fees from Remuneration from Oxford University Press as editor-in-chief of *Human Reproduction Open*, personal fees from Editor and contributing author, *Reproductive Medicine for the MRCOG*, Cambridge University Press. The remaining authors declare no conflict of interest in relation to the work presented.

**TRIAL REGISTRATION NUMBER:**

Not applicable.

WHAT DOES THIS MEAN FOR PATIENTS?

Collecting eggs (oocyte retrieval) through the vagina is crucial for attempting conception using IVF and ICSI. As it is a painful procedure, many different options for pain relief have been studied. The aim of this study was to assess the quality and usefulness of research on pain relief during oocyte retrieval. To do this, we searched for relevant studies that had been published in scientific and medical journals. Our assessment was based on a set of important features of how the studies had been carried out (methodological characteristics) and a set of clinically relevant characteristics. The methodological characteristics we assessed were features that are important for research in all fields, such as the process of randomly assigning participants to study groups, and the calculation of how many participants were needed per group. The clinical characteristics were features specifically relevant to research on pain relief during oocyte retrieval, such as whether pain and patient satisfaction were measured, and when and how these were measured.

The most important methodological characteristics were reported inadequately in 30–43% of the included studies. Methods used for measuring clinical characteristics varied greatly. For example, 10 different methods were used for measurement of pain and 12 for measurement of satisfaction. This makes a comparison of different studies difficult, and it raises the question of how relevant some of the outcomes are for patients. Therefore, we provide recommendations for the design of future studies on this topic.

## Introduction

Transvaginal oocyte retrieval is crucial in the process of IVF and ICSI. As it is a painful procedure, many different options for pain relief have been studied. A recently updated Cochrane review on conscious sedation and analgesia (CSA) for women undergoing oocyte retrieval concluded that no single method was superior to another (Kwan *et al.*, 2018). The overall quality of the evidence was assessed to be low or very low, however, mainly owing to poor reporting. Another limitation was the broad heterogeneity of interventions used, as well as outcomes measured, limiting the possibility of accurately comparing and applying the results.

Poor quality of randomized controlled trials (RCTs) and their reporting has been described in all fields (Clarke and Williamson, 2016; Moher *et al.*, 2016; Glasziou and Chalmers, 2018; Duffy *et al.*, 2020).

This may lead to an over- or underestimation of an effect of an intervention, and it hinders inclusion and meta-analysis in systematic reviews. Considering the waste of valuable research money and the ethical implications of poorly conducted and interpretable research, it is important that all trials are methodologically strong, and that reporting is transparent and complete.

The aim of this review is to provide an overview of the methodology of RCTs on pain relief during oocyte retrieval conducted thus far, to assess their validity and usefulness. Furthermore, we provide recommendations for methodology and reporting, to prevent further heterogeneity and to improve the quality of future trials on this topic.

## Materials and methods

### Literature search

A systematic search was performed on 20 July 2020 in the following databases: Cochrane Library, CENTRAL CRSO, MEDLINE, Embase, PsycINFO, CINAHL, ClinicalTrials.gov, WHO ICTRP, Web of Science, Portal Regional da BVS and Open Grey. The search strategy for each individual database is provided in the Supplementary Data. Additionally, the reference lists of systematic reviews on this topic were checked for relevant publications. Titles and abstracts were independently screened by two reviewers, E.T.I.A.B and H.G. All full texts of RCTs on analgesia or anaesthesia during oocyte retrieval in which pain or satisfaction were an outcome measure were included. Only full texts of RCTs on pain relief during oocyte retrieval were included to ensure that detailed information on methodology could be retrieved.

### Data extraction

We evaluated a series of methodological characteristics known to affect the validity of RCTs. These were based on a critical appraisal tool designed by the Joanna Briggs Institute (Tufanaru *et al.*, 2017). Furthermore, we assessed reporting of clinical characteristics deemed relevant for this specific topic. The characteristics evaluated are summarized in Table I. Two authors (E.T.I.A.B and H.G.) independently assessed occurrence and reporting of all characteristics listed below. Any disagreement was resolved by a third author (J.W.v.S.).

**Table I:** A list of methodological and clinical characteristics assessed in all included articles on pain relief during oocyte retrieval.

List of characteristics assessed
Methodological characteristics: Randomization and allocation concealment. Blinding of participants, personnel and outcome assessors. Sample size calculation, compared with final sample size. Primary outcome measure and secondary outcome measures. Whether all treatment groups were treated similarly, aside from the intervention of interest. Whether a (CONSORT) flowchart was included. Whether baseline characteristics in the different treatment groups were similar. Intention-to-treat analysis. Whether follow-up was complete, and if not, whether the differences between the groups were adequately described. Selective reporting of outcomes.
Clinical characteristics: Measurement of intra- and postoperative pain, and if so, exact timing, and which scale was used. Measurement of patient satisfaction, and if so, the scale used. Registration and definition of side effects, complications and pregnancy rate. Inclusion of infertility diagnosis, number of follicles and/or oocytes in baseline characteristics. Whether there were any significant results for the outcomes of pain or satisfaction.

To provide a trend of occurrence and reporting of characteristics over time, we divided the included RCTs into three groups based on the year of publication, and stratified the data according to these groups. Time trends are expressed as a percentage.

Although the purpose of this review is to assess overall methodology, and not to perform a meta-analysis of results, we did note and summarize outcomes of each studied intervention.

## Results

### Included articles

**Figure 1.**
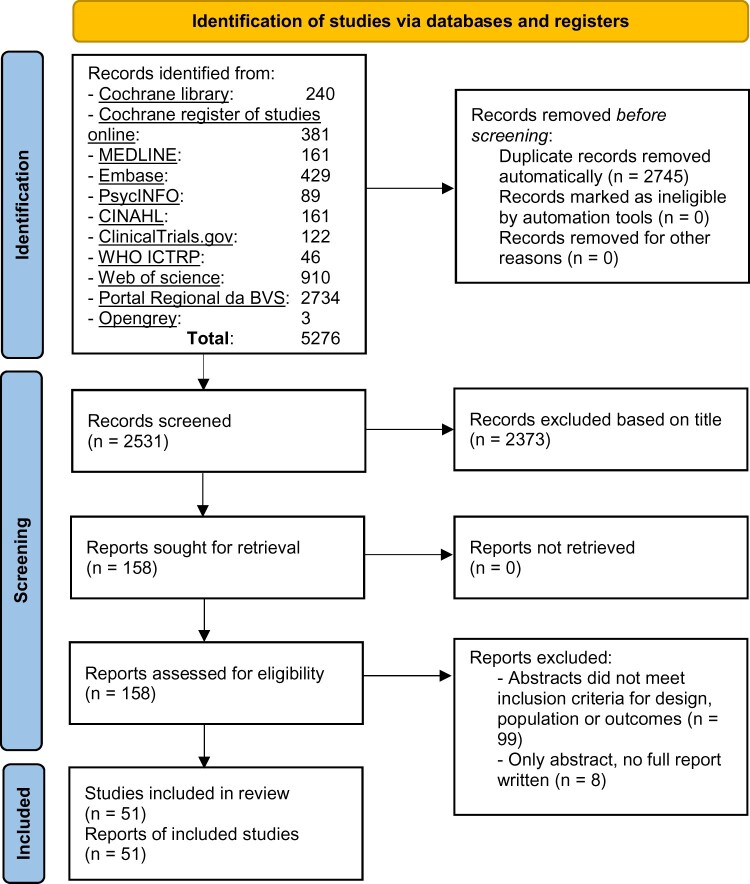
PRISMA flow diagram for a review of research on pain relief during oocyte retrieval for IVF/ICSI.

A total of 2531 titles, after automatic removal of duplicates, were screened. Fifty-one RCTs were included in this review, as listed and described in Table II. Figure 1 shows the selection of articles for this review. The included RCTs were published between 1990 and 2020. They were divided into three groups based on year of publication to identify time trends: one group of 13 RCTs published in the 1990s (1990–1999), one group of 25 RCTs published in the 2000s (2000–2009) and a third group of 13 RCTs published between 2010 and 20 July 2020 (2010–2020).

**Table II:** This table lists the 51 randomized controlled trials included in this review on research on pain relief during oocyte retrieval, the interventions compared per trial and whether they studied pain, satisfaction or both.

Reference	Interventions studied	Outcome(s)
Atashkhoii *et al.*, 2006	CSA and PCB vs. CSA and placebo PCB	Pain and satisfaction
Ben-Shlomo *et al.*, 1992	CSA intravenously and PCB vs. CSA intramuscularly with PCB	Pain and satisfaction
Ben-Shlomo *et al.*, 1999	CSA vs. GA	Pain and satisfaction
Bhattacharya *et al.*, 1997	Patient-controlled CSA vs. physician-controlled CSA	Pain and satisfaction
Casati *et al.*, 1999	Different drug regimens for GA	Pain and satisfaction
Cerne *et al.*, 2006	CSA with pre-ovarian block vs. CSA with PCB	Pain
Chen *et al.*, 2012	Dolantin with EA vs. Dolantin alone	Pain
Cook *et al.*, 1993	Patient-controlled CSA vs. physician-controlled CSA	Satisfaction
Corson *et al.*, 1994	CSA with PCB vs. CSA with placebo PCB vs. CSA alone	Pain
Coskun *et al.*, 2011	Different drug regimens for target-controlled infusion CSA	Pain and satisfaction
Coskun *et al.*, 2017	Target-controlled infusion CSA with different fentanyl boluses	Pain
Elnabtity and Selim, 2017	Different drug regimens for CSA with PCB	Pain and satisfaction
Gejervall *et al.*, 2005	CSA with PCB vs. EA with PCB	Pain and satisfaction
Godoy *et al.*, 1993	CSA intramuscularly with different PCB drug regimens	Pain
Guasch *et al.*, 2005	GA vs. spinal anaesthesia vs. CSA with PCB in two different drug regimens	Pain and satisfaction
Gunaydin *et al.*, 2007	CSA vs. CSA with PCB	Pain and satisfaction
Hong *et al.*, 2007	Different drug regimens for GA	Pain
Humaidan and Stener-Victorin, 2004	CSA with PCB vs. EA with PCB	Pain
Humaidan *et al.*, 2006	PCB with different frequencies of EA	Pain
Kailasam *et al.*, 2010	CSA with NSAID postoperatively vs. CSA without NSAID postoperatively	Pain
Lai *et al.*, 2020	Different drug regimens for CSA	Pain and satisfaction
Lier *et al.*, 2015	Patient-controlled CSA vs. physician-controlled CSA	Pain and satisfaction
Lok *et al.*, 2002	Patient-controlled CSA vs. physician-controlled CSA	Pain and satisfaction
Ma *et al.*, 2008	Different drug regimens for CSA	Pain
Manica *et al.*, 1993	Different drug regimens for spinal anaesthesia	Pain
Martin *et al.*, 1999	Spinal anaesthesia with fentanyl vs. spinal anaesthesia alone	Pain
Matsota *et al.*, 2012	CSA vs. GA	Satisfaction
Meng *et al.*, 2008	CSA vs. CSA with EA	Pain
Meng and Wang, 2009	CSA vs. CSA with EA	Pain
Morue *et al.*, 2018	CSA with ketamine vs. CSA with placebo	Pain and satisfaction
Ng *et al.*, 1999	CSA with PCB vs. CSA with placebo PCB vs. CSA alone	Pain
Ng *et al.*, 2000	CSA with different dosages of PCB	Pain
Ng *et al.*, 2001	CSA with PCB vs. placebo CSA with PCB	Pain and satisfaction
Ng *et al.*, 2002	CSA with PCB and pethidine intramuscularly vs. CSA with PCB and saline intramuscularly (placebo)	Pain and satisfaction
Ng *et al.*, 2003	CSA with different drug regimens for PCB	Pain
Öcal *et al.*, 2002	CSA vs. CSA with NSAID	Pain
de Oliveira Júnior *et al.*, 2016	Different drug regimens for GA with or without PCB	Pain and satisfaction
Ozturk *et al.*, 2006	CSA vs. CSA with PCB	Pain and satisfaction
Ramsewak *et al.*, 1990	CSA vs. placebo CSA	Pain
Ramzy *et al.*, 2001	Postoperative lignocaine in subcortical region of the ovary vs. placebo vs. none	Pain
Saleh *et al.*, 2012	Different drug regimens for GA	Pain and satisfaction
Sarikaya *et al.*, 2011	Different drug regimens for CSA	Satisfaction
Sator-Katzenschlager *et al.*, 2006	Patient-controlled CSA with auricular EA vs. with auricular acupuncture without stimulation vs. with placebo acupuncture	Pain and satisfaction
Stener-Victorin *et al.*, 1999	CSA with PCB vs. EA with PCB	Pain
Stener-Victorin *et al.*, 2003	CSA with PCB vs. EA with PCB	Pain
Thompson *et al.*, 2000	Patient-controlled CSA vs. physician-controlled CSA	Pain and satisfaction
Tummon *et al.*, 2004	Analgesia intravenously with vaginal gel vs. analgesia intravenously with PCB	Pain
Zaccabri *et al.*, 2001	Nasal midazolam with PCB vs. nasal midazolam with vaginal gel	Pain
Zelcer *et al.*, 1992	Patient-controlled CSA vs. physician-controlled CSA	Pain and satisfaction
Zhang *et al.*, 2013	NSAID with acupuncture vs. NSAID with placebo acupuncture	Pain
Zhao *et al.*, 2017	GA with premedication vs. GA with placebo premedication	Pain

CSA, conscious sedation and analgesia; EA, electro-acupuncture; GA, general anaesthesia; NSAID, non-steroidal anti-inflammatory drug; PCB, paracervical block.

Of the 51 included articles, four were written in Chinese, one in French, one in Spanish and one in Turkish. The remaining 44 were written in English. All articles included an English abstract. In three RCTs, pain was not measured, but they were included because satisfaction rate was measured.

### Methodological characteristics

**Figure 2.**
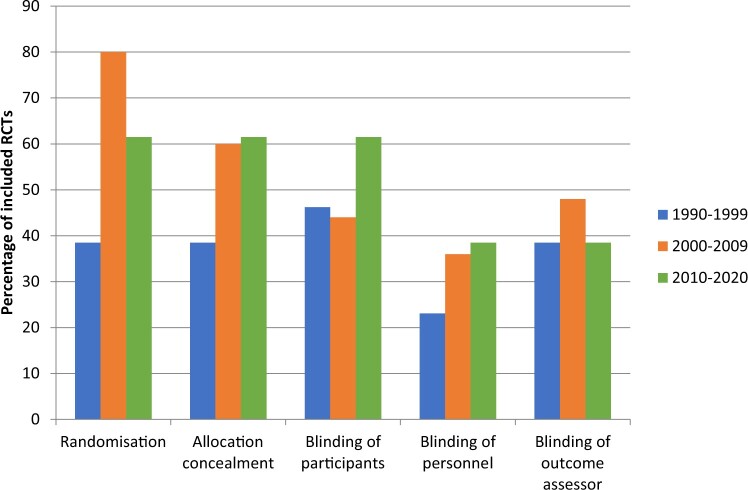
**Frequency of reported methodological characteristics in randomized controlled trials on pain relief during oocyte retrieval.** Data are presented in three 10-year intervals to display trends. RCT, randomized controlled trial.

**Figure 3.**
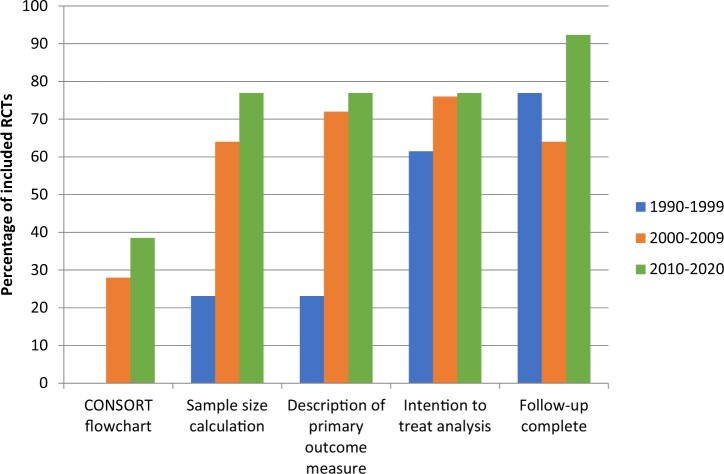
**Frequency of additional reported methodological characteristics in randomized controlled trials on pain relief during oocyte retrieval.** Data are presented in three 10-year intervals to display trends. RCT, randomized controlled trial.

The findings on methodological and clinical characteristics are summarized in Table III. Trends of methodological characteristics are displayed in Figs 2 and 3, respectively.

**Table III:** The methodological and clinical characteristics that were assessed in all RCTs on pain relief during oocyte retrieval.

	Yes—N (%)	No—N (%)	Unclear—N (%)
**Methodological characteristics**			
True randomization	33 (64.7%)	1 (2.0%)	17 (33.3%)
Allocation concealment	28 (54.9%)	3 (5.9%)	20 (39.2%)
Blinding			
Of participants	25 (49.0%)	21(41.2%)	5 (9.8%)
Of personnel	17 (33.3%)	24 (47.1%)	10 (19.6%)
Of outcome assessor	22 (43.1%)	4 (7.8%)	25 (49.0%)
Outcome measures defined	32 (62.7%)	19 (37.3%)	N/A
Sample size calculation	29 (56.9%)	21 (41.2%)	1 (2.0%)
Calculated sample size reached	25 (86.2%)	4 (13.8%)	N/A
Follow-up complete	38 (74.5%)	13 (25.5%)	N/A
Adequately described if not?	9 (69.2%)	4 (30.8%)	N/A
Intention-to-treat analysis	37 (72.5%)	11 (21.6%)	3 (5.9%)
CONSORT flowchart	12 (23.5%)	39 (76.5%)	N/A
Identical treatment protocols aside from intervention of interest	46 (90.2%)	5 (9.8%)	N/A
Selective reporting	2 (3.9%)	49 (96.1%)	N/A
Baseline characteristics similar	43 (84.3%)	3 (5.9%)	5 (9.8%)
**Clinical characteristics**			
Measurement of pain	48 (94.1%)	3 (5.9%)	N/A
Intraoperative pain	39 (76.5%)	11 (21.6%)	1 (2.0%)
Assessed during procedure*	21 (53.8%)	18 (46.2%)	N/A
Postoperative pain*	31 (60.8%)	19 (37.3%)	1 (2.0%)
Satisfaction	25 (49.0%)	26 (51.0%)	N/A
Baseline characteristics			
Infertility diagnosis	24 (47.1%)	27 (52.9%)	N/A
Number of follicles or oocytes	26 (51.0%)	25 (49.0%)	N/A
Pregnancy rate	28 (54.9%)	23 (45.1%)	N/A
Definition provided	13 (46.4%)	6 (21.4%)	9 (32.1%^†^)
Side effects	39 (76.5%)	12 (23.5%)	N/A
Definition provided	34 (87.2%)	5 (12.8%)	N/A
Complications	20 (39.2%)	25 (51.0%)	N/A
Definition provided	15 (80.0%)	5 (20.0%)	N/A

* Nearly every article provided a different definition for the exact timing of assessment of pain. This could, therefore, not be specified in more detail in this table.

^†^ The definition for pregnancy rate was regarded ‘unclear’ when a description such as ‘clinical pregnancy’ was provided, but it was not specified how and when this was assessed.

#### Randomization and allocation concealment

Randomization was reported in all articles, but it was described insufficiently in one-third of the articles (17/51; 33%). Thirty-three (65%) of the RCTs described a low-risk method of randomization. In most cases, this entailed a computer-generated list (20/33; 61%) or a random numbers table (8/33; 24%). A low-risk method of allocation concealment was described in a little over half of the articles (28/51; 55%), most often with sealed, opaque envelopes, in 26/28 RCTs (93%). As shown in Fig. 2, reporting of randomization and allocation concealment has markedly increased since the 1990s, but reporting of randomization was 20% higher in the 2000s than in the period from 2010 until now.

#### Blinding

Nearly half of the included RCTs reported that participants were blinded to group allocation (25/51; 49%). Blinding of personnel was reported in one-third of the RCTs (17/51; 33%). Blinding of the outcome assessor occurred in 22/51 RCTs (43%). An upward trend can be seen in blinding of participants and personnel over the past decades, with an increase of over 10%, although blinding of personnel still occurred in only a little over one-third of the RCTs published in the last decade (Fig. 2). The rate of outcome assessor blinding has remained stable over the decades.

#### Outcome measures

Outcome measures were reported in 32/51 articles (63%). Fifteen different primary outcome measures were reported, which are listed in Table IV. The rate of reporting of the primary outcome measure has more than tripled since the 1990s, and remained stable at ∼75% of the RCT in the past 18 years.

**Table IV:** The primary outcome measures in all included articles, and the frequency of occurrence, in RCTs on pain relief during oocyte retrieval.

Primary outcome	Number of included RCTs (%)
Intraoperative pain	14 (27.5%)
Postoperative pain	1 (2.0%)
Recovery	
Time to ambulation/time to void/time to discharge	1 (2.0%)
Length of PACU stay	2 (3.9%)
STAI of well-being 60 min after procedure	1 (2.0%)
Satisfaction	1 (2.0%)
Pregnancy rate	3 (5.9%)
Incidence of respiratory impairment	1 (2.0%)
Incidence of dizziness or drowsiness	1 (2.0%)
Drug related	
Average propofol dose	1 (2.0%)
Plasma concentration of remifentanil	2 (3.9%)
Hormone related	
ACTH/cortisol and prolactin concentration in follicular fluid	1 (2.0%)
Plasma cortisol concentration	1 (2.0%)
Number of follicles punctured	1 (2.0%)
Fertilization rate of oocytes	1 (2.0%)
Not stated	20 (39.2%)

One article reported two primary outcomes: the STAI and postoperative pain. A sample size calculation was performed for both outcomes. For other articles, which stated having more than one primary outcome measure, but performed a sample size calculation on only one, the primary outcome measure was considered to be the outcome for which the study was powered.

ACTH, adrenocorticotropic hormone; PACU, postanaesthesia care unit; RCT, randomized controlled trial; STAI, state-trait anxiety inventory.

#### Sample size calculation

A sample size calculation was described in just over half of the included RCTs included (29/51; 57%). A strong upward trend of inclusion of a sample size calculation can be seen over the past decades, from 23% in the 1990s to 77% in the 2010s (Fig. 3).

#### Follow-up and analysis

Follow-up was complete in 38/51 (75%) of the included RCTs, and data were analysed according to the intention-to-treat (ITT) principle in 37/51 articles (73%). This was not always explicitly described, but was assumed based on tables and group sizes. A CONSORT flowchart was included in 12/51 RCTs (24%). Follow-up was most often complete in the RCTs published in the 2010s (92%) and least often in the 2000s (64%). Between the 1990s and the 2000s, use of an ITT analysis increased by ∼15%, and it has remained stable since then at ∼75%. As expected, we can see an upward trend for inclusion of a CONSORT flowchart, from 0% in the 1990s to 38% in the 2010s (Fig. 3).

#### Protocols

Treatment groups were not treated identically other than the intervention of interest in 5/51 RCTs (10%). Differences between treatment protocols included the administration of premedication or a paracervical block (PCB) aside from the intervention of interest, and extra clinical visits.

Protocols were publicly registered for 4 of the 51 included RCTs (8%). Small discrepancies in secondary outcome measures were observed between the protocol and the article in two of these (50%). Upon comparison of methods and results reported in the published articles, selective reporting was found to have occurred in an additional 2/51 articles (4%).

### Clinical characteristics

**Figure 4.**
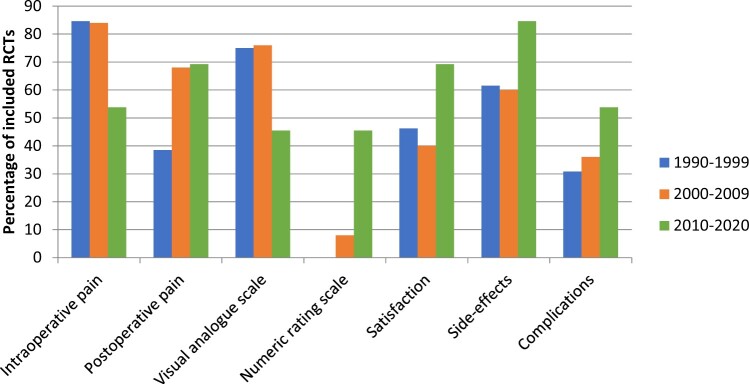
**Frequency of reported clinical characteristics in randomized controlled trials on pain relief during oocyte retrieval.** Data are presented in three 10-year intervals to display trends. RCT, randomized controlled trial.

Trends of clinical characteristics are displayed in Fig. 4.

#### Pain and satisfaction

In the 48 RCTs that measured pain, 10 different scales were used. The most used pain scale was the visual analogue scale (VAS), either from 0 to 10 or 0 to 100. A VAS was used in 33/48 articles (69%). The second most used pain scale was the 11-point numeric rating scale (NRS), on which patients score the pain between 0 and 10. NRS was used in 7/48 articles (15%). All scales used for pain measurement are displayed in Table V. The timing of measurement of pain varied greatly. In 21/39 of the articles that reported intraoperative pain (54%), it was measured during the procedure, whereas 18/39 articles reported remembered intraoperative pain (46%). The timing of postoperative pain measurement varied from directly after the procedure to days after. Forty-one percent of the RCTs that measured pain did not adequately define the timing of the measurement (16/39).

**Table V:** Reported scales for pain measurement in RCTs on pain relief during oocyte retrieval.

Scale for pain measurement	Number of RCTs (%)
Visual analogue scale (0–10 or 0–100)	33 (68.8%)
Numeric rating scale (0–10 or 0–100)	7 (14.6%)
3-level scale	1 (2.1%)
4-level scale	5 (10.4%)
5-level scale	2 (4.2%)
McGill Pain Questionnaire	3 (6.3%)
McGill Pain Questionnaire short form	1 (2.1%)
Pain-rated index (6-level scale)	1 (2.1%)
Multiple scales*	4 (8.3%)

Ten different scales were used. Scales with the same number of levels were grouped together in this table but were considered different when significantly different descriptors were used for the levels.

* These all include the visual analogue scale (n = 3) or the Numeric rating scale (n = 1) and a version of the McGill pain questionnaire. One study also used the pain-rated index.

RCT, randomized controlled trial.

The VAS was the most used method in the first two decades of publications. In the 2010s, the NRS was used as often as the VAS. Intraoperative pain was measured in significantly more articles in the first two decades studied here, than in the 2010s, in which it was measured in only 54% of the included RCTs. Measurement of postoperative pain has increased over time.

Satisfaction was measured in nearly half of the articles (25/51; 49%). The most used method was a 4-level scale (9/25, 36%), although many variations of descriptors per level were given in the RCTs that used it. Eleven other methods were described, which are displayed in Table VI. Willingness to undergo the procedure again under the same type of analgesia or sedation was asked in 7/25 RCTs (28%). Satisfaction was measured most often in the past decade, with a rate of 69%, compared to 46% in the 1990s (Fig. 4).

**Table VI:** The different methods used for measurement of satisfaction in RCTs on pain relief during oocyte retrieval.

Method of measurement of satisfaction	Number of RCTs (%)
2-level scale	2 (8.0%)
3-level scale	2 (8.0%)
4-level scale	9 (36.0%)
7-level Likert scale	1 (4.0%)
8-item questionnaire (CSQ-8)	1 (4.0%)
11-level scale (0–10)	3 (12.0%)
Visual analogue scale (0–100)	1 (4.0%)
Percentage	1 (4.0%)
Overall satisfaction (undefined)	3 (12.0%)
Self-made questionnaire	1 (4.0%)
Interview (undefined)	1 (4.0%)
Willingness to undergo again	7 (28.0%)

In the 25 articles that assessed satisfaction, 12 different methods were used for its measurement. Even when the same number of levels for a scale was used, many different descriptors were used. The question of willingness to undergo the procedure with the same method of analgesia was always combined with another scale.

CSQ, client satisfaction questionnaire; RCT, randomized controlled trial.

#### Baseline characteristics

Baseline characteristics were reported in all RCTs. It was unclear whether baseline characteristics were similar in nearly 10% of the RCTs (5/51). Most included RCTs reported age and weight as baseline characteristics. Infertility diagnosis was reported in 24/51 RCTs (47%), and the number of follicles available or oocytes retrieved were reported in 26/51 (51%).

#### Treatment outcomes, side-effects and complications

Pregnancy rate was reported in a little over half the included RCTs (28/51, 55%). The definition of pregnancy rate varied greatly, from urinary or plasma hCG to clinical or ongoing pregnancy assessed between 4 and 16 weeks’ gestational age. Live birth rate (LBR) was assessed in two RCTs (4%). Over half of the RCTs (15/28; 53%) did not provide an adequate definition. Whether pregnancy was assessed per cycle or per embryo transfer was unclear in 29% of the RCTs (8/28). It was assessed per embryo transfer in 12 RCTs (43%), and per cycle in 10% (3/28). Various RCTs also reported fertilization, cleavage and implantation rate, or number of embryos, usually without definitions.

Thirty-nine of the 51 RCTs (77%) reported having assessed side-effects. In five of these (13%), a definition of side-effects assessed was lacking. Assessment of complications was reported in 20/51 RCTS (39%). Again, these were not defined in five (20%). The rate of reporting of side-effects and complications has increased over the past decades, from 61% to 85%, and from 31% to 54%, respectively (Fig. 4).

### Interventions and significant results

A great variation of interventions has been compared in the included RCTs. Some RCTs compared different drugs and doses, whereas most RCTs compared different interventions, or the addition of an intervention, such as electro-acupuncture or a PCB. In 40/51 RCTs (78%), a statistically significant difference was found between the intervention and control group in intra- or postoperative pain, patient satisfaction, occurrence of side-effects or complications or pregnancy rate.

CSA was compared to placebo in one RCT, which showed a significantly higher intraoperative pain in the placebo group, confirming the value of pain relief during oocyte retrieval (Ramsewak *et al.*, 1990).

Five RCTs were conducted to study the effect of different dosages of sedatives or analgesics (Manica *et al.*, 1993; Ng *et al.*, 2000, 2003; Coskun *et al.*, 2011; Sarikaya *et al.*, 2011). None of these trials showed a significant benefit of higher dosages in pain scores or patient satisfaction. In one RCT, a higher dose of remifentanil was associated with a significant increase in apnoea, and need for mechanical ventilation. Different combinations of agents and administration routes for CSA, general anaesthesia (GA) or spinal anaesthesia were compared in 12 RCTs (Godoy *et al.*, 1993; Casati *et al.*, 1999; Martin *et al.*, 1999; Öcal *et al.*, 2002; Guasch *et al.*, 2005; Hong *et al.*, 2007; Ma *et al.*, 2008; Saleh *et al.*, 2012; Coskun *et al.*, 2017; Elnabtity and Selim, 2017; Morue *et al.*, 2018, Lai *et al.*, 2020), of which 10 (83%) showed a significant benefit for one of the combinations used. A formal comparison of these outcomes is beyond the scope of this review, but it is important to realize that use of different agents and administration routes of the same category of sedatives or analgesics may need to be studied further to find the optimal combination in this population.

Nine RCTs were included in which acupuncture or electroacupuncture (EA) were compared to other interventions (Stener-Victorin *et al.*, 1999, 2003; Humaidan and Stener-Victorin, 2004; Gejervall *et al.*, 2005; Sator-Katzenschlager *et al.*, 2006; Meng *et al.*, 2008; Meng and Wang, 2009; Chen *et al.*, 2012; Zhang *et al.*, 2013). In the four RCTs in which EA and a PCB were compared to CSA and a PCB, a benefit was seen more often for CSA than for EA in terms of pain and satisfaction. When the addition of (E)A to CSA was compared to CSA alone, or CSA with placebo acupuncture, (E)A did seem to have a beneficial effect. However, in four of these nine RCTs, treatment groups were not treated identically aside from the intervention of interest. One RCT was conducted to compare different frequencies for EA (Humaidan *et al.*, 2006), in the form of a fixed frequency and a mixed frequency: this showed similar intra- and postoperative pain scores in both groups, but significantly less nausea intraoperatively in the fixed frequency group.

The effect of addition of a PCB to another intervention was studied in six RCTs (Corson *et al.* 1994; Ng *et al.*, 1999; Atashkhoii *et al.*, 2006; Ozturk *et al.*, 2006; Gunaydin *et al.*, 2007; de Oliveira Júnior *et al.*, 2016). In five of these, intra- or postoperative pain was studied, showing a benefit for addition of a PCB in four RCTs (Corson *et al.* 1994; Ng *et al.*, 1999; Atashkhoii *et al.*, 2006; de Oliveira Júnior *et al.*, 2016). Satisfaction was studied in four RCTs, which showed a benefit for the PCB group in two RCTs (Atashkhoii *et al.*, 2006; Gunaydin *et al.*, 2007). Two RCTs compared vaginal gel to a PCB (Zaccabri *et al.*, 2001; Tummon *et al.*, 2004). Intraoperative pain was significantly lower in the PCB group in both RCTs.

The effect of injection of lidocaine in the vaginal wall and near the ovary, described as a pre-ovarian block (Cerne *et al.*, 2006), was studied in two included RCTs (Ramzy *et al.*, 2001; Cerne *et al.*, 2006). This was performed preoperatively and compared to a PCB in one RCT, and postoperatively after GA in the other. The injection of lidocaine did not produce a significant benefit in terms of pain.

## Discussion

This review focuses on the methodology of all RCTs on pain relief in oocyte retrieval. The key methodological characteristics were inadequately reported in 33–43% of the included RCTs, as shown in Table III. This is an unfortunate type of waste, as, even though a study may be executed well, inadequate reporting diminishes the efforts involved. Over time, reporting has improved for all characteristics except randomization. This downward trend may be explained by the fact that four articles in the 2000–2010 group had the same first author, who reported very adequately. Clearly, there is still plenty of room for improvement.

Furthermore, a broad heterogeneity is revealed in the studied outcome measures. This hinders the generalizability and comparability of the evidence. We discuss the limitations by characteristic and provide recommendations for future research. For reporting, we recommend use of the CONSORT statement (Schulz *et al.*, 2010).

### Methodological characteristics

#### Randomization and allocation concealment

While all articles reported randomization, it was described inadequately in one-third of the articles. Allocation concealment was unclear or inadequate in nearly half of the articles. Randomization and allocation concealment are crucial to prevent selection bias.

#### Blinding

Blinding in any form occurred in less than half of the included RCTs. In the case of subjective outcome measures, non-blinded trials show an exaggeration of the intervention effect when compared to blinded trials (Wood *et al.*, 2008). Considering the subjective nature of pain, likely to be influenced by anxiety and expectations, blinding should always be endeavoured.

Although the complexity of blinding of patients is dependent on the studied intervention, it was possible in 25 RCTs included in this review, for example by administering saline to a placebo group. When it is not possible to blind clinicians, for example when studying acupuncture, the protocol should include standardized moments or assessments to minimize performance bias owing to individual decisions, such as the administration of additional analgesia. If pain is assessed verbally, a non-blinded outcome assessor might ask about pain scores in a suggestive manner, or record outcomes differently (Wood *et al.*, 2008). Someone who is not involved in the procedure could be used as an outcome assessor. If this is not feasible, these methods should be protocolled and reported, for example by using a script.

#### Outcome measures

Pain relief is a clinical, patient-centred topic. When the aim of the RCT is to improve the patient experience, the primary outcome measure should be a patient-centred outcome, such as intra- or postoperative pain, or patient satisfaction. Primary outcome measures such as concentration of analgesics or hormones in plasma are not in accordance with a clinical purpose, as they do not provide information about the effectiveness of an intervention. Secondary outcome measures should also be defined, and should include treatment outcomes, side-effects and complications.

#### Sample size calculation

Forty-one percent of the included RCTs did not report a sample size calculation. It is therefore likely that a proportion of the results are incorrect because of Type II errors (Altman, 1980). In the case of pain as a primary outcome measure, the minimal clinically relevant difference should be decided on when planning the study size. Consensus on what minimal difference is clinically relevant has not yet been reached, although it has been studied for the VAS and the NRS in varying populations (Farrar *et al.*, 2003; Lee *et al.*, 2003; Grilo *et al.*, 2007; Danoff *et al.*, 2018). Eleven included RCTs with pain as a primary outcome adequately reported a sample size calculation, for which six different definitions were used for the minimal difference. In their Cochrane review, Kwan *et al.* (2018) describe that a two-point difference on a 0–10 VAS could represent a clinically important difference. Cepeda *et al.* (2003) showed that a change of 20% on the NRS is considered a minimal improvement postoperatively. Based on these results, it seems reasonable to expect that the minimal clinically relevant difference in pain scores is 20% on the VAS and NRS.

#### Follow-up and analysis

If randomization is timed well, a complete follow-up and use of an ITT-analysis are feasible in research on pain relief during oocyte retrieval. In 27.5% of the included trials, however, an ITT-analysis was inadequately described, or not performed. Most patients were excluded from analysis because of side-effects, scheduling problems or impaired compliance. Some of these are likely to have been influenced by the intended intervention, and are statistically as well as clinically relevant. In these cases, performing an ITT-analysis, as well as a per-protocol analysis, provides useful information on the applicability of the intervention.

#### Protocols

In 10% of the RCTs, treatment protocols between groups were not identical aside from the intervention of interest. In these studies, it is unclear what the effect of the studied interventions were, and what the effects of the additional protocol differences were. This causes further distortion when the results are compared in meta-analyses, and is, therefore, a big shortcoming. Selective reporting was identified in 8% of the RCTs, but a publicly accessible protocol was available for only 4/51 of the included RCTs (8%): it is therefore likely an underrepresentation.

### Clinical characteristics

#### Pain and satisfaction

Two major issues were identified in the measurement of pain in the included trials: 10 different scales were used, and the timing of measurement varied greatly. The VAS and NRS are the most common scales. While the VAS is most often used in all medical fields, some patient categories prefer the NRS (Hjermstad *et al.*, 2011; Bourdel *et al.*, 2015; Chiarotto *et al.*, 2019). This is the case for non-native speakers, elderly patients and patients with trauma and other impairments (Hjermstad *et al.*, 2011), groups that are unlikely to be relevant in large numbers in research on oocyte retrieval. The 0–100 VAS has been argued to be more precise, as it is a continuous scale with 101 levels, but there seems to be little gain in precision with more than seven options (Bandalos and Enders, 1996; Preston and Colman, 2000). Given that most trials to date have used the VAS, it would be useful to continue using this scale so past results can be compared. Most studies have shown a high correlation between VAS and NRS, but others have shown discrepancies (Downie *et al.*, 1978; Karcioglu *et al.*, 2018; Breivik *et al.*, 2000). The comparison of two different scales is never the golden standard, but comparison of these two seems less problematic than with other scales.

The ambiguity of the timing of postoperative pain measurement is likely to influence the applicability of the evidence so far (Kwan *et al.*, 2018). A number of sedatives and analgesics used are known to cause amnesia, and the overall success of treatment might alter a patient’s view of the procedure experience. Therefore, it is unreliable to pool pain scores, measured in a time frame ranging from directly after the procedure to days after, under one heading of postoperative pain. The same can be said about intraoperative pain measured during or after the procedure. Zaccabri *et al.* (2001) measured pain intraoperatively as well as postoperatively, as remembered pain. This is an interesting outcome when the intervention can alter remembered pain.

Satisfaction was measured in nearly half the articles, revealing high overall satisfaction rates. Of the 22 RCTs that measured both pain and satisfaction, a significant difference between groups was seen for both pain and satisfaction in only 5/22 RCTs (22.7%). Whether global satisfaction is a meaningful outcome in health care is under discussion, as satisfaction about the overall success of a treatment overrides the discrimination of satisfaction with specific aspects of it (Wensing and Elwyn, 2003; Leffler *et al.*, 2015). This was shown in satisfaction about procedural sedation by Leffler *et al.* (2015), and is relevant in the population undergoing oocyte retrieval, as overall success in terms of oocytes collected may override distress caused by the pain of the procedure (Kwan *et al.*, 2018). Therefore, to discriminate between satisfaction with pain relief specifically and the treatment overall, it is advisable to use validated questionnaires, such as the PROcedural Sedation Assessment Survey (PROSAS) (Leffler *et al.*, 2015), the Patient Satisfaction with Sedation Instrument (PSSI) (Vargo *et al.*, 2009), or the Iowa Satisfaction with Anesthesia Scale (ISAS) (Dexter *et al.*, 1997). Willingness to undergo the procedure again with the same analgesia method is an interesting addition to other scales, as it provides an answer to a very patient-centred question.

#### Baseline characteristics

All RCTs reported baseline characteristics, but the items varied greatly. A correlation has been shown between pain during oocyte retrieval and age, number of follicles punctured or oocytes retrieved, preoperative anxiety, duration of the procedure and, in some studies, BMI and number of preceding oocyte retrievals (Corson *et al.*, 1994; Tummon *et al.*, 2004; Cerne *et al.*, 2006; Gejervall *et al.*, 2007; Frederiksen *et al.*, 2017). It is possible that certain infertility diagnoses, such as endometriosis, are associated with higher pain scores. Inclusion of these baseline measurements would provide a valuable comparison of the groups, as well as more insight into the relation between pain and patient characteristics.

#### Treatment outcomes, side-effects and complications

While 76.5% of the RCTs reported having assessed side-effects, complications and/or treatment outcomes, many did not provide definitions for those outcomes. Side-effects and complications associated with the studied types of pain relief, as well as the most common effects of oocyte retrieval, should be assessed.

Generally, the most important treatment outcome in fertility care is pregnancy rate, or LBR. LBR was used in only one RCT (Stener-Victorin *et al.*, 1999). Others were not powered for this outcome, and definitions of pregnancy varied greatly. In the context of this research topic, assessing LBR is rarely feasible as it is time-consuming. Furthermore, it is unlikely to be affected by the method of pain relief. If assessment of LBR is not feasible, the viable pregnancy rate confirmed by ultrasound is the most representative alternative.

### Interventions

The difficulty in research on pain relief is that endless combinations of analgesics and sedatives, their corresponding doses and routes of administration are possible. For example, although CSA is used as a common description, this can be performed with many different combinations and dosages of drugs, and even the aim of CSA might differ per trial, varying from light to deep sedation. This heterogeneity in interventions also inhibits generalizability of the results. This cannot truly be prevented, because all different interventions and drug combinations are of interest, but the effect of this heterogeneity can be limited by treating both groups equally, except for the intervention of interest, and by using relevant outcome measures in all RCTs.

### Future research

Heterogeneity in outcome measures is a major issue in research on this topic, and has been identified as a cause of research waste in all fields (Glasziou and Chalmers, 2018). A proposed solution is the development of core outcome sets (COS). This is an increasingly used, consensus-based tool in research, defined as an agreed standardized set of outcomes that should be measured and reported (Williamson *et al.*, 2017; Gargon *et al*., 2019). COS are expected to lead to higher-quality trials, to reduce the heterogeneity between trials and to improve the comparability of the results. Furthermore, COS could be used for clinical audits and comparison between clinics (Williamson *et al.*, 2017). A COS has recently been developed for infertility research (Duffy *et al.*, 2020), but research on pain relief during oocyte retrieval demands a different set of outcomes. Although we provide recommendations for outcomes that we believe should be included in future trials on this subject, we have not gone through the process of formally developing COS. Aside from experts’ views, patients’ views are also included in the development of a COS, which might provide useful additional insight for future research.

Recovery on the days after the procedure would also be an interesting, patient-centred outcome and might be influenced by the method of analgesia used. A validated questionnaire, such as the quality of recovery-40 questionnaire, would provide insight into this aspect of the patient experience (Gornall *et al.*, 2013).

In addition, cost-effectiveness may be an important factor for patients in their decision for methods of pain relief, given that ART is not completely reimbursed by health insurance in most countries. It is, therefore, recommended to include a cost-effectiveness analysis in new trials.

### Recommendations for designing future trials

Based on our findings, evidence-based guidelines on conduct and reporting of RCTs, and expert opinion, we provide a series of recommendations for designing future trials of pain relief during oocyte retrieval. These are provided in Table VII.

**Table VII:** Recommendations for designing future trials on analgesia during oocyte retrieval.

Recommendations for future trials
Randomization and allocation concealment: We recommend adherence to the CONSORT guideline for transparent reporting on this topic. Blinding: Blinding of patients and staff should always be endeavoured, because of the subjective nature of the outcome measures of pain and satisfaction. When interventions do not have feasible placebo alternatives, performance bias can be prevented by clear protocols, in which conduct and, for example, justification for dosage changes are registered. It is recommended to use a blinded outcome assessor. Outcomes: First and foremost, outcomes reflecting the patient experience are recommended because of the patient-centred nature of this topic. Intraoperative pain is the preferred primary outcome measure. This should be measured during the procedure, or immediately after, about experienced pain during the procedure. Postoperative pain should be measured at the same time for all patients, and the timing should be described precisely. Although there is no consensus on the preferred scale for pain measurement, most RCTs have used the VAS scale, and we recommend continuing this trend, until further research shows otherwise. Satisfaction is a relevant outcome in means of patient-centred care, but does not seem a valuable primary outcome due to limited discrimination. Use of validated questionnaires for satisfaction and patient experience with sedation and analgesia is recommended. Side effects and complications associated with the interventions should be assessed and defined. Pregnancy rates should be included for assessment of safety of the intervention. Definitions should be clearly described, including gestational age and method of assessment. Most clinically relevant are viable pregnancy rate confirmed by ultrasound, and live birth rate. Whether it was assessed per embryo transfer or per cycle should also be reported. Recovery after oocyte retrieval has rarely been studied. A validated questionnaire such as the quality of recovery-40 questionnaire would provide insight in this aspect of the patient experience. Sample size: For sample size calculation for a study with pain as a primary outcome, the minimal clinically relevant difference should be considered to be at least 20% on an 11-point scale. The sample size depends on the mean pain score expected in the control group. Follow-up and analysis: For transparency about the pathway patients took from counselling to analysis, we recommend use of the CONSORT flowchart. ITT analysis is the most fitting method of analysis in this field. Selective reporting: Most journals currently require a study protocol to have been published before the RCT is considered for publication. It is imperative to have defined outcome measures before starting the RCT. Population: All women undergoing oocyte retrieval can be included in research on pain relief. A description of the population should include age, BMI, infertility diagnosis, including presence of endometriosis, number of follicles punctured and/or oocytes retrieved, and the duration of the procedure. Additionally, pre-operative measurement of anxiety is recommended. Interventions: There seems no need for the more invasive general anaesthesia to be studied any longer. Other than the intervention of interest, treatment groups should be treated in the same way. Cost-effectiveness: It is recommended to include a cost-effectiveness analysis in new trials.

ITT, intention-to-treat; RCT, randomized controlled trial.

## Conclusion

The RCTs performed to date on pain relief during oocyte retrieval have been of low-quality owing to various methodological limitations. Furthermore, the possibilities for accurate comparison and application of the evidence obtained are limited by the broad heterogeneity of interventions studied, as well as outcomes measured.

In this methodological review, the key limitations of reporting of methodological and clinical characteristics in research on pain relief for women undergoing oocyte retrieval were identified. Recommendations are given for designing future trials to help avoid more unproductive research of low quality being conducted, and to improve the comparability and generalizability of future RCTs.

## Supplementary data

Supplementary data are available at *Human Reproduction Open* online.

## Data availability

The data underlying this article will be shared on reasonable request to the corresponding author.

## Authors’ roles

E.T.I.A.B., S.B., R.W. and J.W.v.S. conceived the idea for this review. E.T.I.A.B. and H.G. performed the literature search, the data extraction and the analysis. E.T.I.A.B. wrote the manuscript, and all authors contributed to revision and approved the final version of the manuscript.

## Funding

None.

## Conflict of interest

S.B. reports being the editor-in-chief of *Human Reproduction Open*. For this manuscript, he was not involved with the handling process within *Human Reproduction Open*, or with the final decision. Furthermore, S.B. reports personal fees from Remuneration from Oxford University Press as editor-in-chief of *Human Reproduction Open*, personal fees from Editor and contributing author, *Reproductive Medicine for the MRCOG*, Cambridge University Press. The remaining authors declare no conflict of interest in relation to the work presented.

## Supplementary Material

hoac006_Supplementary_Data
