# Supplementary material for: Trends in research on pain relief during oocyte retrieval for IVF/ICSI: a systematic, methodological review
Source: Hum Reprod Open. 2022 Feb 16;2022(1):hoac006. doi: 10.1093/hropen/hoac006 (PMC8868119; doi:10.1093/hropen/hoac006)
Supplement: hoac006_Supplementary_Data [file hoac006_supplementary_data.docx]

**Supplementary Data**

**Search strategy for a systematic review of pain relief during oocyte retrieval.**

**All searches were performed on July 20th 2020**

1. **Cochrane Library**

Keywords CONTAINS "oocyte" or "oocyte aspiration" or "oocyte collection" or "oocyte donors" or "oocyte pick‐up" or "oocyte pickup techniques" or "oocyte retrieval" or "follicular aspiration" or "follicle aspiration" or "donor egg cycles" or "donor oocytes" or "Aspirating ICSI" or "Aspiration" or Title CONTAINS "oocyte" or "oocyte aspiration" or "oocyte collection" or "oocyte donors" or "oocyte pick‐up" or "oocyte pickup techniques" or "oocyte retrieval" or "follicular aspiration" or "follicle aspiration" or "donor egg cycles" or "donor oocytes" or "Aspirating ICSI" or "Aspiration"

AND

Keywords CONTAINS "conscious sedation" or "sedation" or "sedatives" or "sedatives, nonbarbituate" or "alprazolam" or "diazepam" or "lorazepam" or "midazolam" or "midolazam" or "oxazepam" or "fentanyl" or "narcotics" or "opioid analgesia" or " opioids" or "bolus" or "antianxiety agents" or "anxiolytic" or "propofol" or "pain relief" or "*Analgesics, Opioid" or "analgesics" or "analgesia" or "anaesthetics" or "anaesthesia" or "acupuncture" or "electroacupuncture" or "pethidine" or Title CONTAINS "conscious sedation" or "sedation" or "sedatives" or "sedatives, nonbarbituate" or "alprazolam" or "diazepam" or "lorazepam" or "midazolam" or "midolazam" or "oxazepam" or "fentanyl" or "fentenyl" or "narcotics" or "opioid analgesia" or "opioids" or "bolus" or "antianxiety agents" or "anxiolytic" or "propofol" or "pain relief" or "*Analgesics, Opioid" or "analgesics" or "analgesia" or "anaesthetics" or "anaesthesia" or "acupuncture" or "electroacupuncture" or "pethidine"

1. **results**
2. **Cochrane register of studies online (CRSO)**

#1 MESH DESCRIPTOR Fertilization in Vitro EXPLODE ALL TREES

#2 MESH DESCRIPTOR Ovarian Follicle EXPLODE ALL TREES

#3 MESH DESCRIPTOR Oocytes EXPLODE ALL TREES

#4 MESH DESCRIPTOR Oocyte Retrieval EXPLODE ALL TREES

#5 MESH DESCRIPTOR Oocyte Donation EXPLODE ALL TREES

#6 MESH DESCRIPTOR Sperm Injections, Intracytoplasmic EXPLODE ALL TREES

#7 (oocyt* adj5 retriev*):TI,AB,KY

#8 (oocyt* adj5 pickup*):TI,AB,KY

#9 (oocyt* adj5 pick up*):TI,AB,KY

#10 (egg* adj5 (retriev* or pick?up* or pick?up*)):TI,AB,KY

#11 (IVF or ICSI):TI,AB,KY

#12 (vitro fertili*):TI,AB,KY

#13 (intracytoplas* adj3 sperm*):TI,AB,KY

#14 (egg* adj2 recover*):TI,AB,KY

#15 (oocyte* adj2 recover*):TI,AB,KY

#16 (follic* adj2 aspirat*):TI,AB,KY

#17 (ovum adj2 aspirat*):TI,AB,KY

#18 (oocyte* adj2 aspirat*):TI,AB,KY

#19 (egg* adj2 aspirat*):TI,AB,KY

#20 (egg* adj2 collect*):TI,AB,KY

#21 (oocyte* adj2 collect*):TI,AB,KY

#22 (ovum adj2 pick?up OR pick?up):TI,AB,KY

#23 (egg* adj2 dona*):TI,AB,KY

#24 (oocyte* adj2 dona*):TI,AB,KY

#25 ((egg* or oocyte*) adj2 donor*):TI,AB,KY

#26 #1 OR #2 OR #3 OR #4 OR #5 OR #6 OR #7 OR #8 OR #9 OR #10 OR #11 OR #12 OR #13 OR #14 OR #15 OR #16 OR #17 OR #18 OR #19 OR #20 OR #21 OR #22 OR #23 OR #24 OR #25

#27 MESH DESCRIPTOR Hypnotics and Sedatives EXPLODE ALL TREES

#28 MESH DESCRIPTOR Conscious Sedation EXPLODE ALL TREES

#29 MESH DESCRIPTOR Narcotics EXPLODE ALL TREES

#30 MESH DESCRIPTOR Fentanyl EXPLODE ALL TREES

#31 MESH DESCRIPTOR Tranquilizing Agents EXPLODE ALL TREES

#32 MESH DESCRIPTOR Anti‐Anxiety Agents EXPLODE ALL TREES

#33 (fentanyl or medazepam):TI,AB,KY

#34 (diazepam or midazolam):TI,AB,KY

#35 (propofol or ketamine or isoflurane):TI,AB,KY

#36 MESH DESCRIPTOR Anesthesia and Analgesia EXPLODE ALL TREES

#37 MESH DESCRIPTOR analgesia EXPLODE ALL TREES

#38 MESH DESCRIPTOR acupuncture analgesia EXPLODE ALL TREES

#39 MESH DESCRIPTOR Electroacupuncture EXPLODE ALL TREES

#40 sedation:TI,AB,KY

#41 (hypnotic* or sedative*):TI,AB,KY

#42 (paracervical block):TI,AB,KY

#43 pethidine:TI,AB,KY

#44 (analgesi* or pain relief):TI,AB,KY

#45 (electro‐acupuncture or electroacupuncture):TI,AB,KY

#46 (anaesthe* or anesthe*):TI,AB,KY

#47 opioid*:TI,AB,KY

#48 alfentanil:TI,AB,KY

#49 (bolus adj2 injection*):TI,AB,KY

#50 #27 OR #28 OR #29 OR #30 OR #31 OR #32 OR #33 OR #34 OR #35 OR #36 OR #37 OR #38 OR #39 OR #40 OR #41 OR #42 OR #43 OR #44 OR #45 OR #46 OR #47 OR #48 OR #49

#51 #26 AND #50

1. **results**
2. **MEDLINE**

1 Fertilization in Vitro/ or Ovarian Follicle/ or Oocytes/

2 exp oocyte donation/ or exp oocyte retrieval/

3 (oocyt$ adj5 retriev$).tw.

4 (oocyt$ adj5 picku$).tw.

5 (egg$ adj5 (retriev$ or picku$)).tw.

6 (IVF or ICSI).tw.

7 (in vitro adj fertili$).tw.

8 (intracytoplas$ adj5 sperm).tw.

9 (egg$ adj2 recover$).tw.

10 (oocyte$ adj2 recover$).tw.

11 (egg$ adj5 (retriev$ or pick u$)).tw.

12 (oocyt$ adj5 pick u$).tw.

13 (follic$ adj2 aspirat$).tw.

14 (ovum adj2 aspirat$).tw.

15 (oocyte$ adj aspirat$).tw.

16 (egg$ adj aspirat$).tw.

17 (egg$ adj2 collect$).tw.

18 (oocyte$ adj2 collect$).tw.

19 (ovum adj2 pickup).tw.

20 (ovum adj2 pick up$).tw.

21 (egg$ adj2 dona$).tw.

22 ((egg or oocyte$) adj2 donor$).tw.

23 (oocyte$ adj donat$).tw.

24 OR/1‐23

25 exp "hypnotics and sedatives"/ or exp alprazolam/ or exp diazepam/ or exp lorazepam/ or medazepam/ or midazolam/ or nitrazepam/ or oxazepam/

26 exp Conscious Sedation/

27 (hypnotic$ or sedative$).tw.

28 exp narcotics/ or exp fentanyl/ or exp tranquilizing agents/ or exp anti‐anxiety agents/

29 (fentanyl or medazepam).tw.

30 (diazepam or midazolam).tw.

31 (propofol or ketamine or isoflurane).tw.

32 exp analgesia/ or exp acupuncture analgesia/ or exp electroacupuncture/

33 sedation.tw.

34 paracervical block.tw.

35 pethidine.tw.

36 (analgesi$ or pain relief).tw.

37 (electro‐acupuncture or electroacupuncture).tw.

38 (anaesthe$ or anesthe$).tw.

39 opioid$.tw.

40 alfentanil.tw.

41 (bolus adj2 injection$).tw.

42 OR/25‐41

43 24 AND 42

44 randomized controlled trial.pt.

45 controlled clinical trial.pt.

46 randomized.ab.

47 placebo.tw.

48 clinical trials as topic.sh.

49 randomly.ab.

50 trial.ti.

51 (crossover or cross‐over or cross over).tw.

52 OR/44‐51

53 exp animals/ NOT humans.sh.

54 52 NOT 53

55 43 AND 54

**161 results**

1. **Embase**

1 exp fertilization in vitro/

2 exp ovary follicle/

3 exp oocyte donation/

4 exp oocyte retrieval/

5 (oocyt$ adj5 retriev$).tw.

6 (oocyt$ adj5 picku$).tw.

7 (egg$ adj5 (retriev$ or picku$)).tw.

8 (IVF or ICSI).tw.

9 (in vitro adj fertili$).tw.

10 (intracytoplas$ adj5 sperm).tw.

11 (egg$ adj2 recover$).tw.

12 (oocyte$ adj2 recover$).tw.

13 (egg$ adj5 (retriev$ or pick u$)).tw.

14 (follic$ adj2 aspirat$).tw.

15 (ovum adj2 aspirat$).tw.

16 (oocyte$ adj aspirat$).tw.

17 (egg$ adj aspirat$).tw.

18 (egg$ adj2 collect$).tw.

19 (oocyte$ adj2 collect$).tw.

20 (ovum adj2 pickup).tw.

21 (ovum adj2 pick up$).tw.

22 (egg$ adj2 dona$).tw.

23 ((egg or oocyte$) adj2 donor$).tw.

24 (oocyte$ adj donat$).tw.

25 OR/1‐24

26 exp sedative agent/ or exp hypnotic sedative agent/ or exp hypnotic agent/

27 exp conscious sedation/

28 (hypnotic$ or sedati$).tw.

29 exp narcotic agent/

30 exp FENTANYL/

31 exp medazepam/

32 (diazepam or midazolam).tw.

33 (propofol or ketamine or isoflurane).tw.

34 exp tranquilizer/

35 exp anxiolytic agent/

36 (fentanyl or medazepam).tw.

37 exp PATIENT CONTROLLED ANALGESIA/ or exp ANALGESIA/ or exp ACUPUNCTURE ANALGESIA/

38 sedati$.tw.

39 paracervical block.tw.

40 pethidine.tw.

41 (analgesi$ or pain relief).tw.

42 (electro‐acupuncture or electroacupuncture).tw.

43 (anaesthe$ or anesthe$).tw.

44 opioid$.tw.

45 alfentanil.tw.

46 (bolus adj2 injection$).tw.

47 OR/26‐46

48 25 AND 47

49 Clinical Trial/

50 Randomized Controlled Trial/

51 exp randomization/

52 Single Blind Procedure/

53 Double Blind Procedure/

54 Crossover Procedure/

55 Placebo/

56 Randomi?ed controlled trial$.tw.

57 Rct.tw.

58 random allocation.tw.

59 randomly allocated.tw.

60 allocated randomly.tw.

61 (allocated adj2 random).tw.

62 Single blind$.tw.

63 Double blind$.tw.

64 ((treble or triple) adj blind$).tw.

65 placebo$.tw.

66 prospective study/

67 OR/49‐66

68 case study/

69 case report.tw.

70 abstract report/ or letter/

71 OR/68‐70

72 67 NOT 71

73 48 AND 72

1. **results**
2. **PsycINFO**

1 exp Reproductive Technology/

2 (oocyt$ adj5 retriev$).tw.

3 (oocyt$ adj5 picku$).tw.

4 (IVF or ICSI).tw.

5 (egg$ adj5 (retriev$ or picku$)).tw.

6 (in vitro adj fertili$).tw.

7 (intracytoplas$ adj5 sperm).tw.

8 (egg$ adj2 recover$).tw.

9 (oocyte$ adj2 recover$).tw.

10 (egg$ or pick u$).tw.

11 (oocyt$ adj5 pick u$).tw.

12 (follic$ adj2 aspirat$).tw.

13 (ovum adj2 aspirat$).tw.

14 (oocyte$ adj aspirat$).tw.

15 (egg$ adj aspirat$).tw.

16 (egg$ adj2 collect$).tw.

17 (oocyte$ adj2 collect$).tw.

18 (ovum adj2 pickup).tw.

19 (ovum adj2 pick up$).tw.

20 (egg$ adj2 dona$).tw.

21 ((egg or oocyte$) adj2 donor$).tw.

22 (oocyte$ adj donat$).tw.

23 or/1‐22

24 exp Sedatives/ or exp Tranquilizing Drugs/ or exp Hypnotic Drugs/

25 exp Alprazolam/

26 exp Midazolam/

27 exp Propofol/

28 exp Fentanyl/

29 exp Opiates/ or exp Analgesia/ or exp Narcotic Agonists/

30 exp Anesthetic Drugs/

31 (hypnotic$ and sedative$).tw.

32 paracervical block$.tw.

33 pethidine.tw.

34 sedati$.tw.

35 (analgesi$ or pain relief).tw.

36 (electro‐acupuncture or electroacupuncture).tw.

37 (anaesthe$ or anesthe$).tw.

38 opioid$.tw.

39 alfentanil.tw.

40 (bolus adj2 injection$).tw.

41 OR/24-s40

42 23 AND 41

1. **results**
   1. **CINAHL**

S1 (MH "Fertilization in Vitro") OR "ivf"

S2 TX ICSI

S3 TX oocyte retrieval*

S4 TX egg pick up

S5 TX oocyte collection*

S6 (MH "Oocyte Donation") OR "oocyte donation"

S7 TX follicle aspiration*

S8 S1 OR S2 OR S3 OR S4 OR S5 OR S6 OR S7

S9 (MM "Conscious Sedation") OR TX"Conscious Sedation"

S10 (MH "Narcotics+ ") OR TX"narcotics" OR (MH "Analgesics, Opioid+")

S11 (MM "Sedation") OR TX "sedation"

S12 TX "pain relief"

S13 TX "bolus injection*"

S14 (MH "Alfentanil") OR TX "alfentanil"

S15 TX fentanyl

S16 TX diazepam or TX midazolam

S17 TX (propofol or ketamine or isoflurane)

S18 (MH "Analgesia")

S19 (MM "Hypnotics and Sedatives+")

S20 TX hypnotic*

S21 TX analgesi*

S22 TX medazepam or TX lorazepam

S23 (MM "Acupuncture Analgesia") OR (MM "Acupuncture Anesthesia") OR (MM "Anesthesia and Analgesia+")

S24 (MM "Patient‐Controlled Analgesia")

S25 (MM "Electroacupuncture")

S26 TX electroacupuncture or TX acupuncture

S27 TX paracervical block*

S28 TX pethidine

S29 TX anaesthe* or anesthe*

S30 TX opioid*

S31 TX bolus N2 injection*

S32 S9 OR S10 OR S11 OR S12 OR S13 OR S14 OR S15 OR S16 OR S17 OR S18 OR S19 OR S20 OR S21 OR S22 OR S23 OR S24 OR S25 OR S26 OR S27 OR S28 OR S29 OR S30 OR S31 129126

S33 S8 AND S32

1. **results**
2. **ClinicalTrials.gov**

(oocyte recovery AND pain) OR (oocyte retrieval AND pain) OR (oocyte aspiration AND pain) OR (oocyte AND analgesia) OR (oocyte AND analgesic) OR (oocyte AND anaesthesia) OR (oocyte AND anesthesia) OR (oocyte AND sedation) OR (oocyte AND acupuncture) OR (oocyte AND block) OR (oocyte AND remifentanil) OR (oocyte AND fentanyl) OR (oocyte AND propofol) OR (oocyte AND pethidine)

1. **results**
2. **WHO ICTRP**

(oocyte recovery AND pain) OR (oocyte retrieval AND pain) OR (oocyte aspiration AND pain) OR (oocyte AND analgesia) OR (oocyte AND analgesic) OR (oocyte AND anaesthesia) OR (oocyte AND anesthesia) OR (oocyte AND sedation) OR (oocyte AND acupuncture) OR (oocyte AND block) OR (oocyte AND remifentanil) OR (oocyte AND fentanyl) OR (oocyte AND propofol) OR (oocyte AND pethidine)

1. **results**
2. **Web of Science**

TI=(oocyte recovery AND pain) OR TI=(oocyte retrieval AND pain) OR TI=(oocyte aspiration AND pain) OR TI=(oocyte AND analgesia) OR TI=(oocyte AND analgesic) OR TI=(oocyte AND anaesthesia) OR TI=(oocyte AND anesthesia) OR TI=(oocyte AND sedation) OR TI=(oocyte AND acupuncture) OR TI=(oocyte AND paracervical block) OR TI=(oocyte AND remifentanil) OR TI=(oocyte AND fentanyl) OR TI=(oocyte AND propofol) OR TI=(oocyte AND pethidine)

1. **results**
2. **Portal Regional da BVS**

(oocyte recovery AND pain) OR (oocyte retrieval AND pain) OR (oocyte aspiration AND pain) OR (oocyte AND analgesia) OR (oocyte AND analgesic) OR (oocyte AND anaesthesia) OR (oocyte AND anesthesia) OR (oocyte AND sedation) OR (oocyte AND acupuncture) OR (oocyte AND block) OR (oocyte AND remifentanil) OR (oocyte AND fentanyl) OR (oocyte AND propofol) OR (oocyte AND pethidine)

1. **results**
2. **OpenGrey**

(oocyte recovery AND pain) OR (oocyte retrieval AND pain) OR (oocyte aspiration AND pain) OR (oocyte AND analgesia) OR (oocyte AND analgesic) OR (oocyte AND anaesthesia) OR (oocyte AND anesthesia) OR (oocyte AND sedation) OR (oocyte AND acupuncture) OR (oocyte AND block) OR (oocyte AND remifentanil) OR (oocyte AND fentanyl) OR (oocyte AND propofol) OR (oocyte AND pethidine)

**3 results**
